# Supplementary figures and images for: Drug-induced activation of integrin alpha IIb beta 3 leads to minor localized structural changes
Source: PLoS One. 2019 Apr 12;14(4):e0214969. doi: 10.1371/journal.pone.0214969 (PMC6461286; doi:10.1371/journal.pone.0214969)

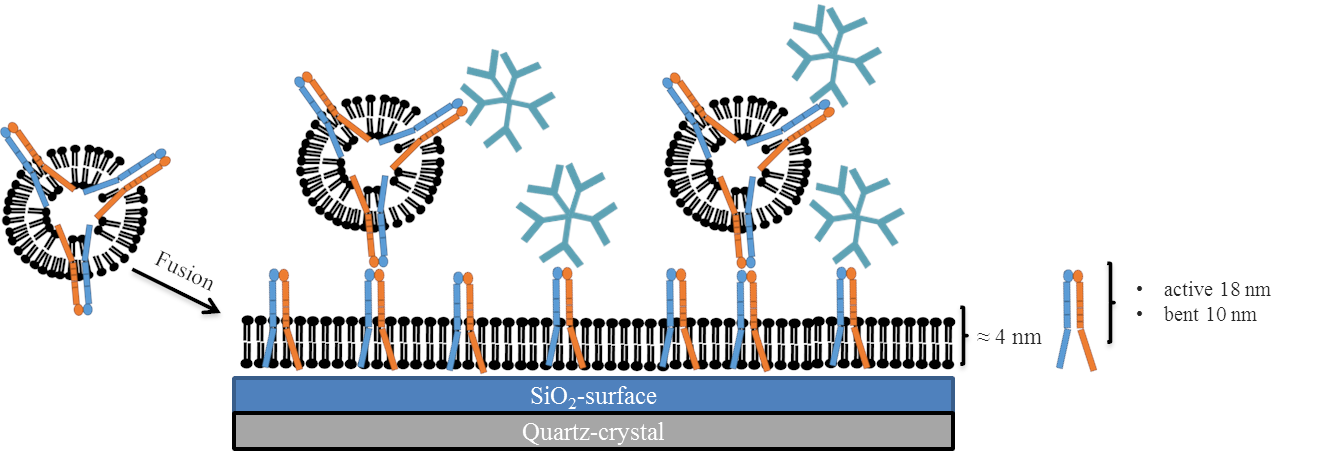

Supplement: S1 Fig — We assume in our QCM-D experiments the fusion of proteoliposomes to the substrate and the formation of a bilayer. Potentially, some proteoliposomes remain on the lipid bilayer surface. Conformation specific antibody PAC-1 (cyan) could bind to activated αIIbβ3 in a bilayer as well as to activated αIIbβ3 in liposomes. (TIF) [file pone.0214969.s001.tif]

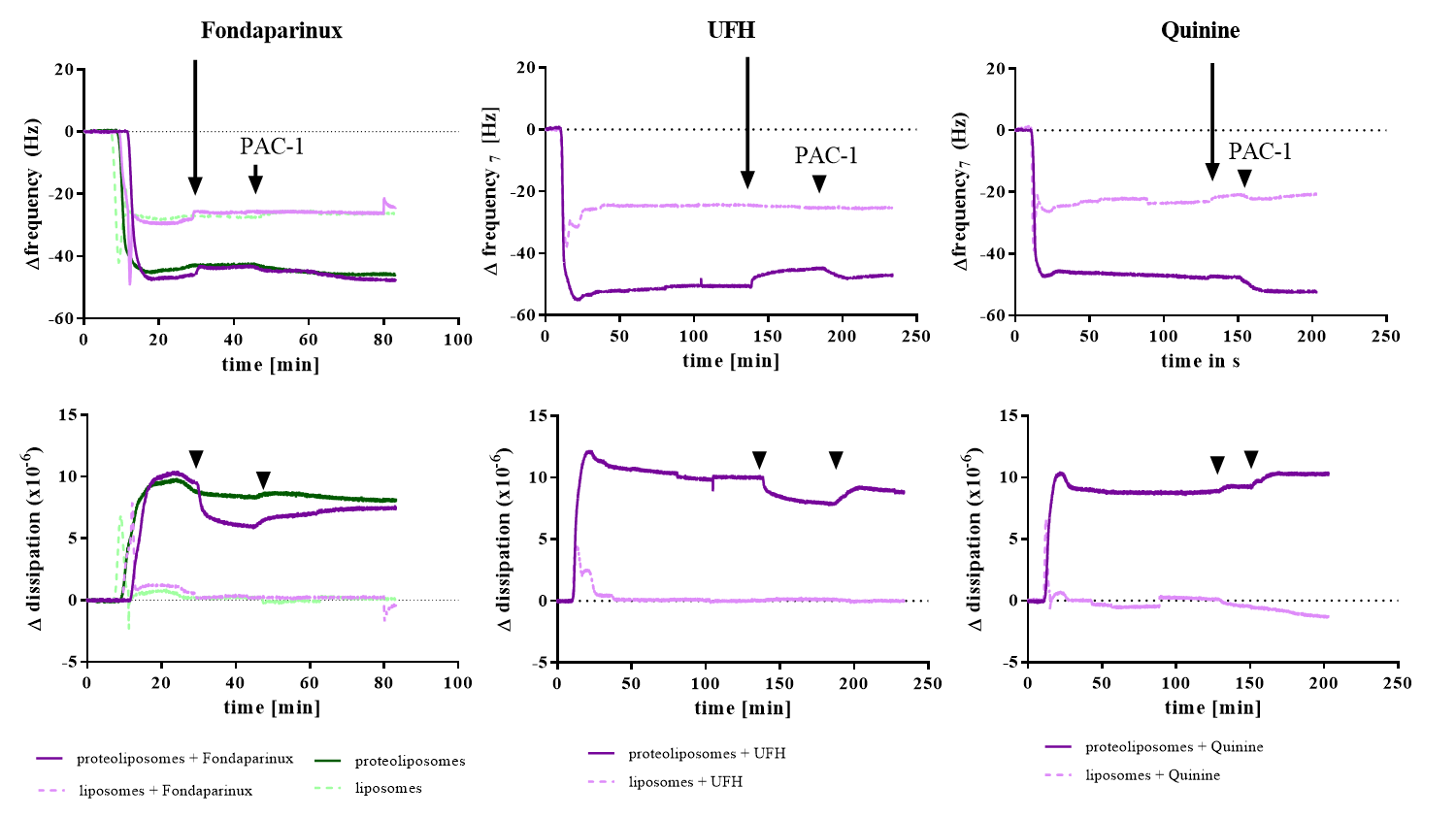

Supplement: S2 Fig — Buffer was injected over the SiO2 sensors and after reaching a baseline, liposomes or proteoliposomes were injected and formation of a bilayer was observed. After a washing step with buffer, the bilayer was treated with the respective drugs (250 μg/mL fondaparinux, 250 μg/mL UFH and 50 μg/mL quinine sulfate), which is indicated by the first arrow and PAC-1 antibody was injected (indicated by the second arrow) followed by rinsing with the respective buffer. (TIF) [file pone.0214969.s002.tif]

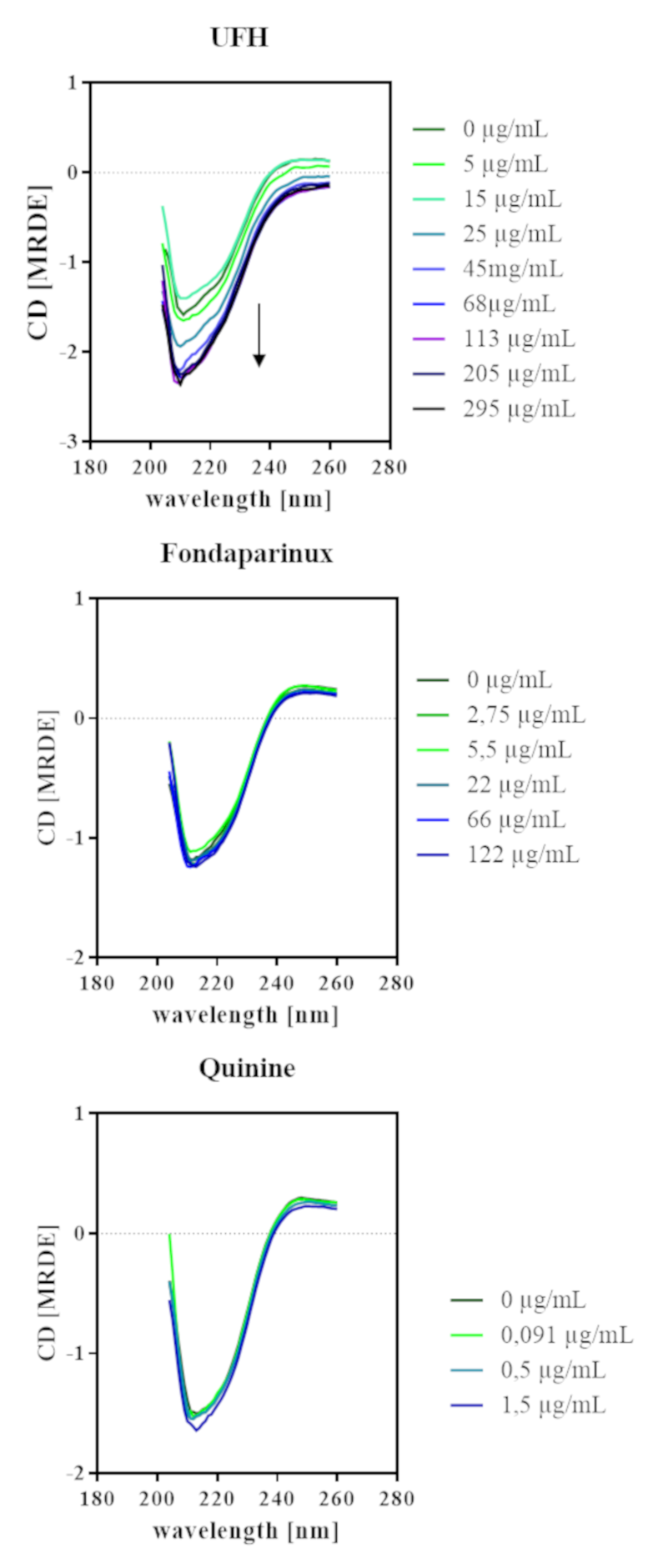

Supplement: S3 Fig — The far-UV region CD spectra of αIIbβ3 reconstituted into liposomes in buffer (dark green), and with increasing concentrations of UFH (top), fondaparinux (middle) and quinine (bottom), respectively. Representative spectra recorded for proteoliposomes with a protein concentration of approximately 0.4 μM in 5 mm path length cuvettes at 37°C are shown. Liposome spectra were subtracted from the respective proteoliposome spectra. (TIF) [file pone.0214969.s003.tif]

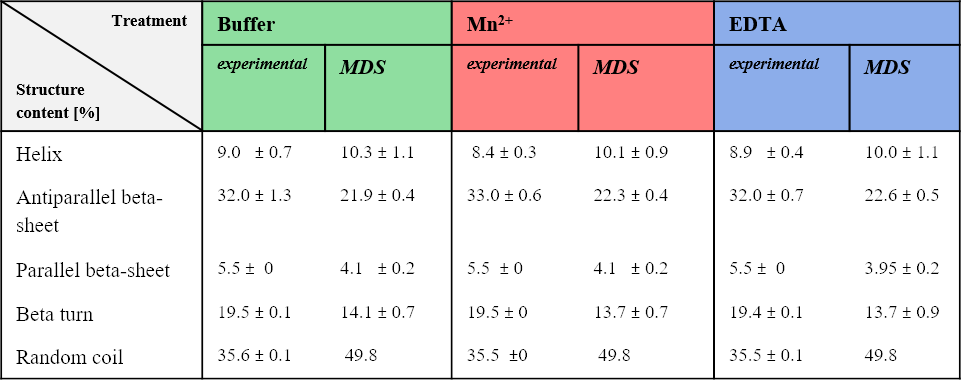

Supplement: S1 Table — Changes in the secondary structure distribution between integrin αIIbβ3 in buffer environment, after addition of 1 mM Mn2+ experimentally or changing the three ions in the MIDAS and ADMIDAS region to Mn2+ via MDS, and after addition of 5 mM EDTA experimentally or removing all structural ions during MDS in the environment. The estimation of the experimental secondary structure content was carried out with the deconvolution of CD spectra using CDNN software. The MDS secondary structure was predicted with CPPTRAJ. (TIF) [file pone.0214969.s004.tif]

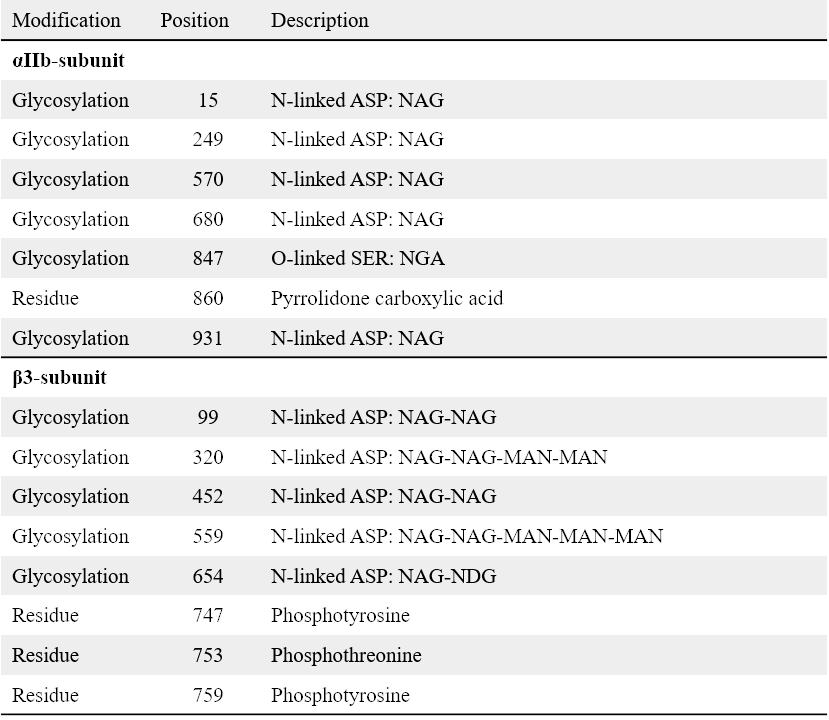

Supplement: S2 Table — Amino acid modifications of the MDS model of integrin αIIbβ3. NDG and NAG are N-Acetylglucosamine in α and β form, respectively, MAN is α-Mannose and NGA is β-N-Acetylgalactosamine. (TIF) [file pone.0214969.s005.tif]

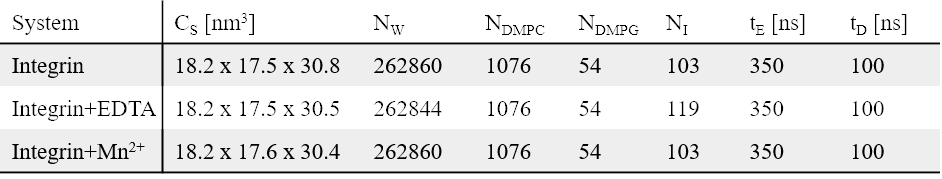

Supplement: S3 Table — System setups for the particular MDS. The first five columns indicate the cell volume Cs, the number of water molecules NW, membrane molecules NDMPC and NDMPG, and ions NI. The last two columns contain the equilibration tE and data collection tD times. (TIF) [file pone.0214969.s006.tif]
